# Atypical Pleural Effusion in an Immunocompetent Patient With Valley Fever: A Case Study and Review of the Literature

**DOI:** 10.7759/cureus.57983

**Published:** 2024-04-10

**Authors:** Sean S Chang, Neil S Hsu, Mariam Khalil, Amy Micheli, Eldo Frezza

**Affiliations:** 1 Surgery, California Northstate University College of Medicine, Elk Grove, USA; 2 Medicine, Colusa Medical Center, Colusa, USA

**Keywords:** fungal infection, pleural coccidioidomycosis, coccidioidomycosis in immunocompetent patient, endemic mycoses, fungal empyema, thoracentesis in valley fever, large pleural effusion, pulmonary coccidioidomycosis, valley fever

## Abstract

Valley fever is a fungal infection, commonly of the lungs, caused by Coccidioides immitis or Coccidioides posadasii. This disease is endemic to the southwestern United States, Central America, and South America. Infected individuals are typically asymptomatic but may develop community-acquired pneumonia. On rare occasions, coccidioidomycosis can present with severe complications in addition to the pulmonary manifestation. In this study, a 58-year-old immunocompetent male presented to the Emergency Department with a cough, night sweats, and pleuritic chest pain. Despite the administration of broad-spectrum antimicrobials, he developed a large right pleural effusion that did not resolve following thoracentesis. Serology was positive for Coccidioides, and the patient was referred to a thoracic surgeon due to persistent effusion. It is rare for patients with coccidiomycosis to develop a large pleural effusion requiring surgical intervention, especially in immunocompetent individuals. This case highlights the importance of monitoring patients with unresolved acute pneumonia in endemic areas and considering Coccidioides as a possible etiology.

## Introduction

Coccidioidomycosis, also known as Valley fever, is a fungal infection endemic to the Western Hemisphere. The fungus primarily grows in the soil of areas with low rainfall, such as the southwestern United States, northern Mexico, and parts of Central and South America [1]. Both human and animal infection results from the inhalation of dust that contains arthroconidia, a type of spore, which is the principal means of dispersal by the Coccidioides species [2]. The most prominent risk factor of this illness is dust exposure within the endemic areas, and the highest incidence is among adults aged 60 and older [3].

The clinical presentation of Valley fever has a broad spectrum, ranging from asymptomatic to severe pneumonic manifestations and disseminated disease. The majority of patients who are symptomatic have pulmonary coccidioidomycosis presenting with fever, cough, chest pain, shortness of breath, and fatigue [4]. In rare cases, the fungal infection may develop into a disseminated form, spreading to the skin, bones, heart, meninges, and other parts of the body. Meningitis is considered to be one of the more severe complications that can lead to hydrocephalus, focal neurological deficits, gait disturbances, and seizures [2]. The diagnosis of Valley fever is typically made through serologic tests, and current therapy involves the administration of amphotericin B and azoles [5].

Pleural effusion is a rare complication of coccidioidomycosis, with few cases being documented. Pleural effusion is estimated to occur in as many as 5%-15% of patients with primary pulmonary coccidioidomycosis [6]. The majority of patients will have a small or moderate effusion that resolves with antifungals, but a large buildup of fluid causing significant clinical symptoms may require thoracentesis [7]. For complex patients, surgical intervention and thoracotomy may be indicated [8]. We present a rare case of coccidioidomycosis with large pleural effusion requiring thoracentesis and surgical intervention.

## Case presentation

**Figure 1:**
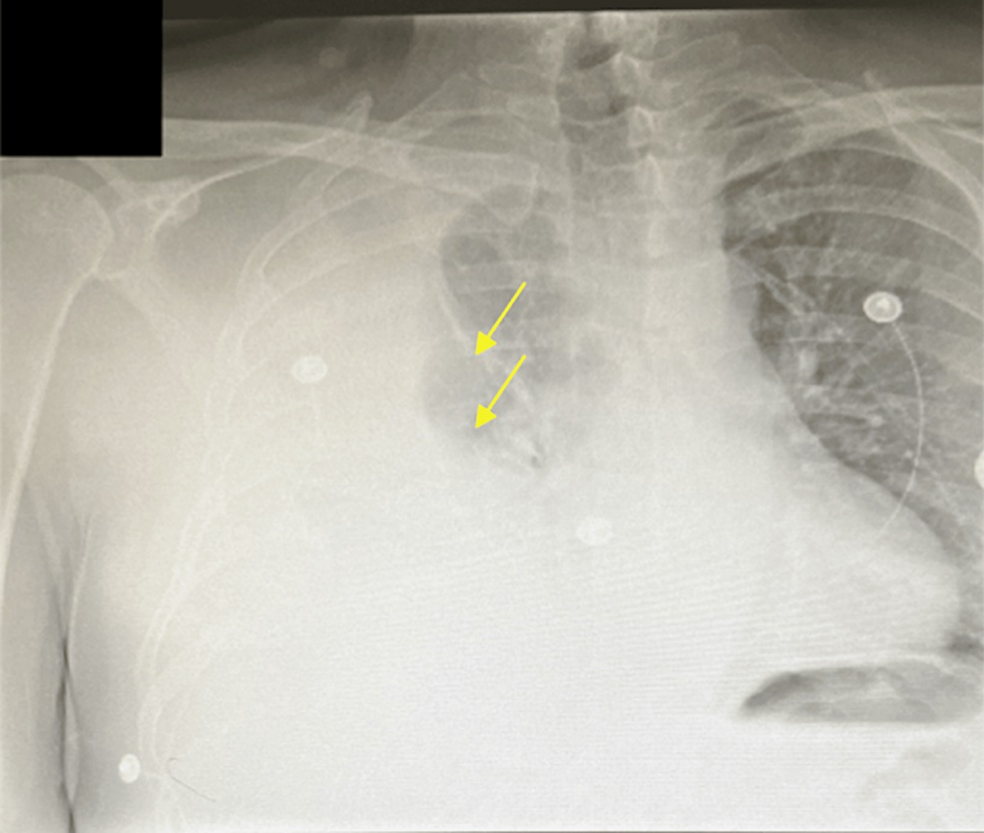
Initial chest X-ray showing pulmonary atelectasis (upper arrow) and a large pleural effusion in the right hemithorax (lower arrow)

A 58-year-old immunocompetent male living in a communal living prison with a history of anxiety and depression presented to the Emergency Department (ED) with night sweats and a cough that he had had for one week. He stated that his symptoms have worsened over the past two days with significant pain upon inspiration in the right lung. Upon evaluation in the ED, he met sepsis criteria with a white blood cell count (WBC) >12 K/uL (4.5-11 K/uL), tachycardia >100 bpm (60-100 bpm), temp >103°F (97-99 °F), and O_2_ sat of 89% (95-100%). The patient responded well to 6 L of supplemental oxygen and was found to have pulmonary atelectasis and a large right pleural effusion on chest X-ray (Figure 1).

**Figure 2:**
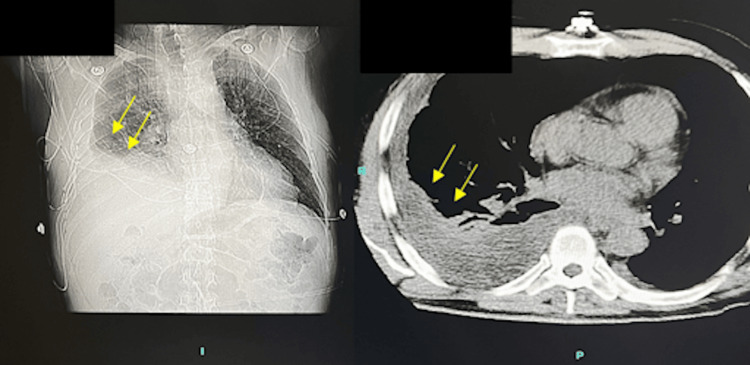
CT scan showing persistent right pleural effusion following administration of antimicrobials CT, computed tomography

Empiric treatment of vancomycin, piperacillin, and tazobactam was started along with fluconazole. The choice of fluconazole treatment on day one was based on the high prevalence of Valley fever during the time of year and the fact that the patient was new to the endemic area. Due to the high suspicion of Valley fever, blood work was done, and the patient was found to be positive for the coccidioides fungus using the coccidioides precipitin test. Additionally, the patient had significant hyponatremia and was given 3% sodium chloride in the ED. Following this, he was given lorazepam for his anxiety that was uncontrolled with his regular anxiety medications. WBC counts were significantly elevated at 26 K/uL (4.5-11 K/uL), and he had an elevated D-dimer of 0.9 mg/L (0-0.5 mg/L). Two days later, the patient was on furosemide 20 mg bid for his pleural effusion. He had a persistent cough, night sweats, and additional fatigue. Supplemental oxygen was decreased from 6 L to 2 L. The next day, the patient reported that he was feeling better, stating that his lung did not feel “restricted” as it did previously. Unfortunately, the following day computed tomography (CT) of his chest indicated persistent right pleural effusion even after antibiotics and diuretics (Figure 2).

**Figure 3:**
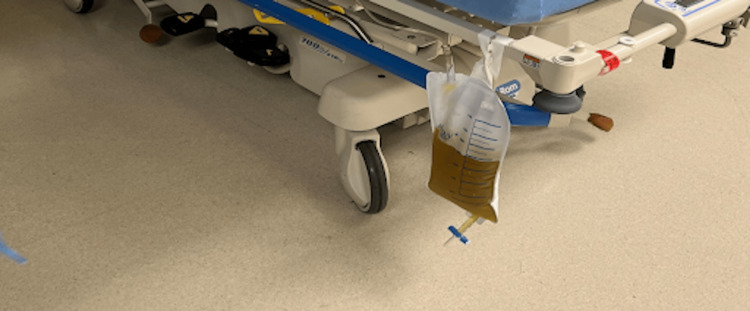
Pleural fluid retrieved from thoracentesis

Thoracentesis was then promptly performed, producing 1200cc of beer-colored clear fluid (Figure 3).

**Figure 4:**
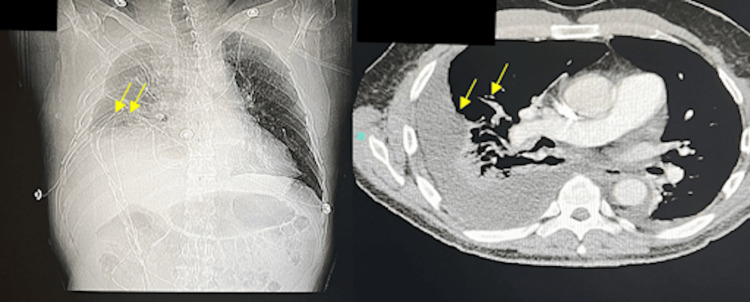
CT scan showing subsequent reaccumulation of pleural fluid following thoracentesis CT, computed tomography

Two hours later, the chest X-ray showed a rapid accumulation of effusion (Figure 4).

The patient was then transferred to another facility with a thoracic surgeon and infectious disease specialist for his worsening complications, where he continued antibiotic therapy and was given a chest tube thoracostomy. Eventually, the patient recovered from the pleural effusion and was discharged from the hospital.

## Discussion

Large pleural effusion is an uncommon manifestation of Valley fever, especially in immunocompetent individuals. The patient's clinical course included initial symptoms suggestive of a lower respiratory tract infection with a clinical diagnosis of pneumonia and sepsis. His chest X-ray showed the presence of a massive right pleural effusion. The subsequent development of a large right pleural effusion posed a diagnostic and therapeutic challenge. Pleural effusions in coccidioidomycosis are infrequently reported, and the majority are small to moderate in size, resolving with antifungal therapy. In this case, the patient's persistent symptoms and worsening chest X-ray necessitated thoracentesis. The unusual nature of this case is underscored by the fact that such manifestations of coccidioidomycosis are typically associated with immunocompromised individuals, whereas the patient in question was immunocompetent.

Coccidioidomycosis is an infection caused by the fungal organisms C. immitis and C. posadasii, which are endemic to the southwestern United States, northern Mexico, and various regions of Central and South America [9]. In the immunocompetent population, an estimated 60% of coccidioidomycosis patients are asymptomatic [10]. Those who are symptomatic often present with pulmonary coccidioidomycosis with flu-like symptoms, including fever, fatigue, cough, shortness of breath, and chest pain. In rare cases (< 1%), coccidioidomycosis can progress to complicated pulmonary disease or present as disseminated disease, commonly infecting the joints, skin, bones, meninges, etc. This is more often seen in immunocompromised patients [11].

In most cases of pulmonary coccidioidomycosis, there is only involvement of the lung parenchyma. Rarely, however, the pleura can become involved, presenting with pneumothorax, empyema, pleuritis, pleural adhesions, and/or pleural effusion [12]. Our patient presented with a large right pleural effusion. Prior studies have estimated that pleural effusions present in 7-20% of symptomatic patients, with 2% of those being large pleural effusions [11]. Merchant et al. further suggest that pleural effusions related to coccidioidomycosis are more likely to present in the left lobe [13]. It is also important to note that pleural involvement of coccidioidomycosis has been suggested to carry a greater risk of disseminated disease [11]. Given that these manifestations of coccidioidomycosis are largely seen in immunocompromised patients, this case is highly unusual. There have only been a small number of similar cases describing immunocompetent coccidioidomycosis patients presenting with a large right pleural effusion [12,14,15]. Furthermore, it is posited that a majority of pleural effusions seen in pleural coccidioidomycosis are due to a result of contiguous infection from the lungs into the pleura as opposed to hematogenous spread [16,17]. This is likely the case for our patient, as he presented with symptoms of intraparenchymal lung involvement, including cough, dyspnea, and inspiratory pain.

## Conclusions

This case study highlights the rare and atypical presentation of coccidioidomycosis, commonly known as Valley fever, in an immunocompetent male. Valley fever is occasionally associated with pulmonary symptoms and pleural involvement. This poses intriguing questions about the pathogenesis of pleural involvement in coccidioidomycosis, the factors influencing its presentation in immunocompetent individuals, and the optimal management strategies for such cases. Additionally, it highlights the fact that despite being immunocompetent, all patients are at risk for pleural effusions and rapidly deteriorating conditions. Due to this, it is essential for prompt evaluation and immediate chest X-rays to be ordered for patients who have not had their symptoms resolved within 48 hours.

Additional studies are warranted to better understand the spectrum of coccidioidomycosis manifestations and refine treatment approaches, especially in the context of uncommon presentations such as large pleural effusion in immunocompetent hosts.
